# Dual inhibition of HSF1 and DYRK2 impedes cancer progression

**DOI:** 10.1042/BSR20222102

**Published:** 2023-01-30

**Authors:** Vasudha Tandon, Rita Moreno, Kira Allmeroth, Jean Quinn, Sandra E. Wiley, Lynden G. Nicely, Martin S. Denzel, Joanne Edwards, Laureano de la Vega, Sourav Banerjee

**Affiliations:** 1 Division of Cellular and Systems Medicine, School of Medicine, University of Dundee, Dundee DD1 9SY, U.K.; 2 Max Planck Institute for Biology of Ageing, Joseph-Stelzmann-Str. 9b, D-50931, Cologne, Germany; 3 Unit of Gastrointestinal Oncology and Molecular Pathology, Institute of Cancer Sciences, College of Medical, Veterinary, and Life Sciences, University of Glasgow, Glasgow, U.K.; 4 Department of Pharmacology, School of Medicine, University of California San Diego, CA 92093, U.S.A.; 5 Altos Labs, Cambridge Institute of Science, Granta Park, Great Abington, Cambridge CB21 6GP, U.K.

**Keywords:** inhibition, myeloma, proteasomes, stress kinases

## Abstract

Preserving proteostasis is a major survival mechanism for cancer. Dual specificity tyrosine phosphorylation-regulated kinase 2 (DYRK2) is a key oncogenic kinase that directly activates the transcription factor heat-shock factor 1 (HSF1) and the 26S proteasome. Targeting DYRK2 has proven to be a tractable strategy to target cancers sensitive to proteotoxic stress; however, the development of HSF1 inhibitors remains in its infancy. Importantly, multiple other kinases have been shown to redundantly activate HSF1 that promoted ideas to directly target HSF1. The eventual development of direct HSF1 inhibitor KRIBB11 suggests that the transcription factor is indeed a druggable target. The current study establishes that concurrent targeting of HSF1 and DYRK2 can indeed impede cancer by inducing apoptosis faster than individual targetting. Furthermore, targeting the DYRK2-HSF1 axis induces death in proteasome inhibitor-resistant cells and reduces triple-negative breast cancer (TNBC) burden in ectopic and orthotopic xenograft models. Together the data indicate that cotargeting of kinase DYRK2 and its substrate HSF1 could prove to be a beneficial strategy in perturbing neoplastic malignancies.

## Introduction

Chromosomal aberrations frequently result in aneuploidy in cancer. This leads to gene dosage imbalances and eventual accumulation of excess misfolded proteins that trigger proteotoxic stress in the neoplastic cells [1]. To survive these aneuploidy-related imbalances, cancer cells rely either on protein degradation via the 26S proteasome or the chaperon-mediated folding pathways through heat-shock factor 1 (HSF1) [2,3]. Therapeutic targeting of key players in the proteotoxic stress pathways have been very successful, especially in haematological malignancies like multiple myeloma and mantle cell lymphoma. Small-molecule proteasome inhibitors such as bortezomib, carfilzomib, and ixazomib have significantly improved the lives of millions of myeloma patients worldwide [4]. Unfortunately, over the years, patients have exhibited refractory and relapsed myeloma with resistance to proteasome inhibitors [5]. Such resistance is caused either due to up-regulation of protein-folding machinery triggered by transcriptional programmes of HSF1 [6,7] or in rare instances, due to point-mutations occurring in the inhibitor-docking subunit PSMB5 of the proteasome in patients [8]. Hence, therapeutic targeting of HSF1 or its upstream regulator(s) could be a novel and potent mechanism to impede cancer progression and chemoresistance. In that sense, HSF1 targetting has been explored resulting in the development of KRIBB11 that directly inhibits HSF1 with low micromolar efficacy [9]. KRIBB11 exhibits cytotoxic effects in various cancer cells in the micromolar range while it also targets tumour burden *in vivo* [10,11]. Since then, HSF1 inhibitor NXP800 has entered Phase I trials for patients with advance cancers (NCT05226507).

Recent works have established a common upstream kinase regulator of the proteasome and HSF1 [12–14]. Dual specificity tyrosine phosphorylation-regulated kinase 2 (DYRK2) is a major regulator of proteostasis that phosphorylates the RPT3 subunit on the 26S proteasome [13] and also Ser320 and Ser326 on HSF1 [14]. Phosphorylation of the 26S proteasome by DYRK2 increases its peptidase activity toward dysfunctional/misfolded proteins [13]. On the other hand, phosphorylation of HSF1 promotes its nuclear translocation and transcriptional function of encoding heat-shock proteins that act as molecular chaperones to assist in protein folding [14]. Indeed, targetting DYRK2 with various small-molecule inhibitors like curcumin, harmine, or LDN192960 can result in cancer reduction both *in vitro* and *in vivo* [15–17]. Specifically, LDN192960 can reduce tumour burden in both multiple myeloma and triple-negative breast cancer (TNBC) models [16]. DYRK2 inhibitors in combination with proteasome inhibitors synergistically induced cytotoxicity [15,16], while LDN192960 alone bypassed bortezomib resistance in myeloma cells and reduced matrigel invasion in TNBC cells [16]. These observations suggest that dual inhibition of DYRK2 and HSF1 could be a beneficial combination in impeding cancer, especially in proteasome inhibitor-resistant models. Indeed, protein levels of DYRK2 positively correlate with active HSF1 levels in TNBC patient tumours and together associates with poor outcome [14]. Importantly, DYRK2 depletion reduces HSF1 transcriptional activity and sensitises TNBC cells to proteotoxic stress [14]. Although DYRK2 is the only kinase reported to phosphorylate HSF1 on both activation sites Ser320 and Ser326 [14], there are other reported redundant kinases which can phosphorylate and activate HSF1 [18]. Additionally, DYRK2 also controls the proteasome. Hence, the effect of dual inhibition of DYRK2 and HSF1 could indeed be additive and needs to be explored in the context of cancer reduction.

In the current study, we explore the link between DYRK2 and HSF1 further, and query whether dual pharmacological inhibition of DYRK2 and HSF1 could induce enhanced cytotoxicity in proteasome inhibitor resistant cells and whether concurrent inhibition or loss-of-activity of DYRK2 and HSF1 ablates cancer progression. We show that targeting the DYRK2-HSF1 axis induces death in proteasome inhibitor-resistant cells and that dual loss of DYRK2 and HSF1 is indeed additive toward reducing TNBC tumour burden in ectopic and orthotopic xenograft models. Thus, dual targetting of HSF1 and its upstream regulator DYRK2 may represent a novel approach to evade drug-resistance, and reduce cancer burden *in vivo*.

## Materials and methods

### Materials

Antibodies used in this study were: anti-Tubulin (Santa Cruz sc-8035), anti-HSF1 (Enzo Life Science ADI-SPA-901-D), anti-cleaved PARP (Cell Signalling 9546S), and anti-DYRK2 (Cell Signalling 8143: for immunoblotting; Abgent AP7534a: for immunohistochemistry).

### General methods

All recombinant DNA procedures, electrophoresis, and immunoblotting were performed using standard protocols. DNA constructs used for transfection were purified from *Escherichia coli* DH5α using Macherey-Nagel NucleoBond® Xtra Maxi kits according to the manufacturer’s protocol. All DNA constructs were verified by DNA sequencing. For shRNA lentivirus production using pLKO1.GFP vector, HEK293T cells were transfected at 80–90% confluency using Lipofectamine 2000 and psPAX2 and pMD2.G packaging vectors. Medium was changed 6–8 h after transfection and supernatant was collected after 72 h. Viral media were passed through a prewetted 0.8-mm PVDF filter (Millipore) and mixed with 8 μg/ml polybrene (Sigma-Aldrich) before being added to recipient MDA-MB-231 cells. Infected GFP-positive population of cells were enriched by flow cytometry and cell sorting using BD FacsJazz. The shRNA sequences used to knock-down DYRK2 have been reported previously (sh1 D2: gggtagaagcggtattaaa & sh2 D2: ggagaaaacgtcagtgaaa) [13]. For qRT-PCR analysis, total RNA from MDA-MB-231 cells were isolated using the NucleoSpin RNA kit (Macherey-Nagel, Bethlehem, PA, U.S.A.). cDNA was synthesised using the iScript kit (Bio-Rad). qRT-PCR analysis was performed using the SYBR® Premix Ex Taq™ II (Takara) on Applied Biosystems 7500 Real-Time PCR System. Data were normalised to corresponding GAPDH levels. Primers used for hGAPDH (Forward: ACATCGCTCAGACACCATG; Reverse: TGTAGTTGAGGTCAATGAAGGG), and hDYRK2 (Forward: TGCATTTTCCTCTCCAGCG; Reverse: ACTGTTGAACCTGGATCTGTC) were purchased from IDT.

### Drug treatment

Harmine (Tocris 5075), LDN-192960 (Sigma-Millipore SML0755), KRIBB11 (Tocris 5480), bortezomib (Selleckchem S1013), carfilzomib (Selleckchem S2853), ixazomib (Selleckchem S2180), and oprozomib (Selleckchem S7049) were dissolved in DMSO at a stock concentration of 10 mM and treatments were carried out as indicated. Curcumin (Sigma-Millipore 08511) was diluted in DMSO at a stock concentration of 5 mM in the dark and prepared fresh prior to each experiment and the excess solution was never stored. Curcumin treatment at a final concentration of 5 µM was always carried out in media containing either 1% bovine serum albumin (BSA) or 10% fetal bovine serum (FBS) to maintain maximum stability and to avoid aggregation [15,19].

### Cell culture

Mammalian cells were all grown in a humidified incubator with 5% CO_2_ at 37°C. HEK293T, MDA-MB-231, and MDA-MB-468 cells were purchased from ATCC and cultured in Dulbecco’s Modified Eagle Media (DMEM, Gibco) supplemented with 10% FBS, 1% L-glutamine, and 1% penicillin and streptomycin. MDA-MB-231 HSF1 knock-out and DYRK2 knock-out cells were generated previously [14]. MM.1S and KMS18 cells were cultured as stated previously [20,21]. Briefly, parental- or bortezomib-resistant MM.1S and KMS18 cells were maintained in RPMI-1640 with 10% FBS and 1% penicillin and streptomycin. Parental- or genome-edited AN3-12 mouse haploid embryonic stem cells were cultured as previously described [21]. In brief, AN3-12 cells were grown in DMEM high glucose (Sigma-Aldrich, St. Louis, MO, U.S.A.) supplemented with 15% FBS, penicillin/streptomycin, glutamine, nonessential amino acids, sodium pyruvate (all Thermo Fisher Scientific, Waltham, MA, U.S.A.), β-mercaptoethanol, and LIF (both Merck Millipore, Darmstadt, Germany) on noncoated tissue culture plates.

### Cell viability and invasion assays

Cell viability assays were carried out with or without 48–72 h treatment of indicated drugs or DMSO control using the CellTiter 96® AQueous Non-Radioactive Cell Proliferation Assay kit (Promega), following manufacturer’s instructions and data were represented as %viability compared with DMSO-treated control. The 3D Matrigel invasion assays were performed using 8 μm pore size transwells coated with Matrigel™ (BD Biosciences) as described previously [22]. The bottom chamber contained normal growth media (DMEM with 10% FBS with or without 5 μM curcumin or 10 μM harmine) as a chemoattractant. MDA-MB-231 cells were seeded into the upper chamber (20000 cells/insert) in DMEM with 1% BSA with or without 5 μM curcumin or 10 μM harmine. After 24 h of culture, cells that migrated through the matrix were quantified using Cyquant following manufacturer’s instructions (Life Technologies).

### HSF1 and DYRK2 expression correlation studies

Expression analysis from single-cell RNA sequencing datasets: This analysis was carried out as stated previously [23]. To understand expression across various cell states of diverse cancers, we queried DYRK2 and HSF1 levels in previously published single-cell RNA sequencing datasets of cancer [24–27] available on the Single Cell portal of Broad Institute, MIT and Harvard, U.S.A. (https://singlecell.broadinstitute.org/single_cell). Data represented with either t-distributed stochastic neighbour embedding (t-SNE) clustering or uniform manifold approximation and projection (uMAP).

Expression change after treatment with a drug combination in clinic: The expression correlation studies were carried out by querying the Champions Oncology proprietary database using Lumin webtool (https://database.championsoncology.com/login/). The Champions PDX bank was derived from human patient tumours prior to (treatment-naïve) or after undergoing standard-of-care therapy (post-therapy). Molecular data (whole-exome and RNA sequencing) from these PDXs were obtained after passaging (2–3 passages) in immunocompromised mice. This gene signature was calculated based on the differential expression of DYRK2 and HSF1 in PDXs obtained from post-therapy vs. treatment-naïve patient tumours. Thus, this signature reflects the correlative HSF1 and DYRK2 gene expression in the indicated tumours after undergoing standard-of-care therapy with the indicated drugs. The individual values are sign-corrected log10 *P*-values of gene expression differences.

Expression correlation with efficacy for an individual drug *in vivo*: This signature reflects the HSF1 and DYRK2 gene expression profile that predicts *in vivo* efficacy to standard of care drugs tested in the Champions Oncology PDX models. This signature was calculated by correlating DYRK2 and HSF1 gene expression across PDX models with efficacy (tumour growth inhibition index) to the standard of care monotherapy drugs described. The individual values are sign-corrected log10 *P*-values of expression difference.

Furthermore, HSF1 and DYRK2 expression correlation was carried out from the cancer genome atlas database using GEPIA2 webtool (http://gepia2.cancer-pku.cn/). The *P*-value and Spearman’s correlation coefficient R were derived from the webtool.

### Animal studies

Mice were housed and maintained at the University of California-San Diego (UCSD) in full compliance with policies of the Institutional Animal Core and Use Committee (IACUC) protocol S03039 approved 31st March 2020. At a maximum tumour volume of 1.2 cm^3^, mice were killed initially under carbon dioxide followed by cervical dislocation.

Ectopic tumour implantation: 6-week-old-female NSG mice (NOD.Cg-Prkdcscid Il2rgtm1Wjl/SzJ; Stock: 005557) were purchased from the Jackson Laboratory. A total of 300000 MDA-MB-231 cells were resuspended in 1:1 slurry with Matrigel™ and subcutaneously injected into the neck of each mouse at *n*=8 mice per cell strain. No anaesthesia was used for the procedure. Tumour dimensions were measured twice per week using a digital caliper and tumour volume was calculated as (length × width^2^)/2. After the indicated days postinjection, mice were killed as stated previously and tumours were excised and weighed. The investigator was not blinded to cell strain allocation during tumour implantation, data collection, and outcome assessment.

Mammary fat-pad orthotopic implantation: A total of 300000 MDA-MB-231 cells were resuspended in 1:1 slurry with Matrigel™ and injected into the #4 mammary fat pad of 8–12 weeks old female J:NU (athymic nude mice; Jackson Laboratory; Stock: 007850) mice under anesthesia at *n*=5 mice per cell strain. Tumour dimensions were measured twice per week using a digital caliper and tumour volume was calculated as (length × width^2^)/2. After the indicated days postinjection, mice were killed as stated previously and tumours were excised and weighed. The investigator was blinded to cell strain allocation during tumour implantation, data collection, and outcome assessment.

### Patient cohort and tissue analysis

A tissue microarray (TMA) was constructed from a cohort (*n*=850) of patients presenting with primary invasive ductal breast cancer at two Glasgow hospitals between 1995 and 1998. The study was approved by the Research Ethics Committee of the West Glasgow University Hospitals NHS trust with consent obtained from all subjects. ER, PR, and HER2 status were assessed on TMAs using IHC as stated previously [14] and *n*=148 samples were identified as triple-negative. Clinicopathological data including age, tumour size, tumour grade, lymph node status, type of surgery, and use of adjuvant treatment (chemotherapy, hormonal-based therapy, and/or radiotherapy) and eventual recurrent tumour progression were retrieved from the routine reports. Tumour grade was assigned according to the Nottingham Grading System. Prior to staining, the TMAs were baked for 30 min then dewaxed by immersion in Histoclear before being rehydrated through a series of alcohols. Heat-induced antigen retrieval was performed in Tris-EDTA buffer pH 9 after which sections were incubated in 3% H_2_O_2_ to exhaust endogenous peroxidases. Nonspecific binding was blocked by incubation with 1.5% horse serum prepared in antibody diluent. DYRK2 antibody (Stratech AP7534a) was diluted in antibody diluent to a concentration of 1:200, applied to the sections, and incubated overnight at 4°C. Appropriated controls were included. Staining was visualised using ImmPRESS™ and ImmPACT™ DAB then counter stained with Harris Haematoxylin before being dehydrated and mounted using DPX. Data collection, analysis, and scoring were carried out as stated previously [14]. DYRK2 expression was categorised as either ‘low’ or ‘high’, in relation to a cutoff that was determined using a ROC curve based on survival, with cancer death as an endpoint.

### Establishing the AN3-12 PSMB5 G183D knock-in cell line

AN3-12 PSMB5 G183D mutant cell line was generated using Crispr/Cas9 similar to the other mutants as reported previously [21]. Briefly, the targetting guide sequences (TCCAGCCATCCTCCCGCACG and TAAGTCAGCTACATTGTCAC) were designed using CRISPOR webtool (http://crispor.org) and were purchased from Sigma-Aldrich. The guide sequences were cloned into the Cas9-GFP expressing plasmid PX458 (Addgene #48138). To generate the *Psmb5* G183D mutant cell line, the plasmids were cotransfected along with the corresponding single stranded DNA repair template (GACAGATACACTACTGTACTTGTCATGTAAATCAGCTACATTATCACTAGACACCCGGATCCAGTCATCCTCCCGGACGTGATAGAGGTTGACTGCCCCTCCGGAGTAGGCATCTCTGTA purchased from Sigma-Aldrich) into AN3-12 cells using Lipofectamine 2000 (Thermo Fisher Scientific). The PAM sites of the guides were mutated in the repair template. Cells were transferred to 10 cm plates 24 h post-transfection and selected with 25 nM bortezomib for 2 weeks. Resistant colonies emerging from single cells were picked and analysed. Positive clones detected by Sanger sequencing were sorted as diploid cells using a FACSAria Fusion sorter prior to further experiments. Following this, we established the sensitivity of the G183D mutant against 10 nM bortezomib, 15 nM carfilzomib, 50 nM ixazomib, and 80 nM oprozomib using using the XTT cell proliferation Kit II (Roche Diagnostics, Basel, Switzerland) after 72 h drug treatment.

### Statistics and data presentation

Details of all statistical tests and multiple comparisons used to derive *P*-value have been detailed in figure legends. All experiments were repeated two to three times with multiple technical replicates to be eligible for the indicated statistical analyses, and representative image has been shown. All results are presented as mean ± SD unless otherwise mentioned. For animal studies, only female NSG and J:NU mice were utilised. For animal studies, statistical power analysis was used to predetermine sample size. Effect size (Cohen’s d) was estimated from smaller pilot experiments using the R package effsize. Power analysis was performed in the R package pwr utilising estimated Cohen’s d, a significance level of 0.05, and power of 0.8. Data were analysed using Graphpad Prism statistical package.

## Results

### DYRK2 and HSF1 expression positively correlates in cancer

**Figure 1:**
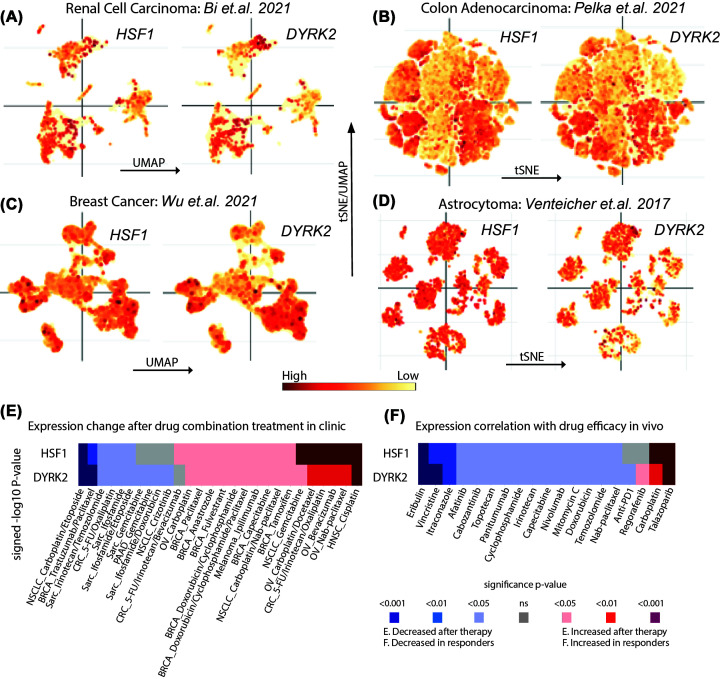
HSF1 and DYRK2 expressions moderately overlap in tumour microenvironment cell states and therapy responses in various cancers Individual RNA expressions of HSF1 and DYRK2 overlayed on the (**A**) UMAP distribution of renal cell carcinoma sc-RNAseq dataset, (**B**) tSNE distribution of colon adenocarcinoma sc-RNAseq dataset, (**C**) UMAP distribution of breast cancer sc-RNAseq dataset, (**D**) tSNE distribution of astrocytoma sc-RNAseq dataset. (**E**) The signed -log10 *P-*value coexpressions of HSF1 and DYRK2 shown in response to indicated drug combination treatment in the respective cancers. (**F**) The signed -log10 *P*-value coexpressions of HSF1 and DYRK2 shown in relation to the response toward individual cancer monotherapies. See also Supplementary Figure S1.

We have previously established that the protein expressions of nuclear DYRK2 and nuclear HSF1 positively correlate with each other in 148 primary patient-derived TNBC samples [14]. To further consolidate this, we explored multiple databases to understand whether DYRK2 and HSF1 expression correlate across diverse cancer datasets [24–27]. We initially investigated recently reported single-cell RNA sequencing (sc-RNAseq) datasets across a few different cancers and queried whether DYRK2 and HSF1 expression overlapped between the heterogenous cell populations within diverse tumours. We observed clear expression overlap between DYRK2 and HSF1 in clustered cell populations of renal cell carcinoma (Figure 1A) and colon adenocarcinoma (Figure 1B) along with breast cancer (Figure 1C). Clustered cell populations in astrocytoma also exhibited a modest overlap of DYRK2 and HSF1 (Figure 1D). Moreover, we saw a positive correlation (Spearman coefficient *R*=0.33) between DYRK2 and HSF1 expression across all cancers in TCGA database (Supplementary Figure S1). Intriguingly, expressions of DYRK2 and HSF1 seemed to be either significantly coup-regulated or codown-regulated in response to standard-of-care clinical chemotherapeutic combination regimens across diverse cancers (Figure 1E). On a similar note, coexpression of DYRK2 and HSF1 seemed to be predictive of positive or negative response to standard-of-care monotherapies in animal PDX models (Figure 1F). Overall, multiple independent databases seem to agree that HSF1 and DYRK2 expressions overlap and correlate in diverse cancer models and also in responses to chemotherapies.

### DYRK2-HSF1 axis promotes TNBC cell survival

**Figure 2:**
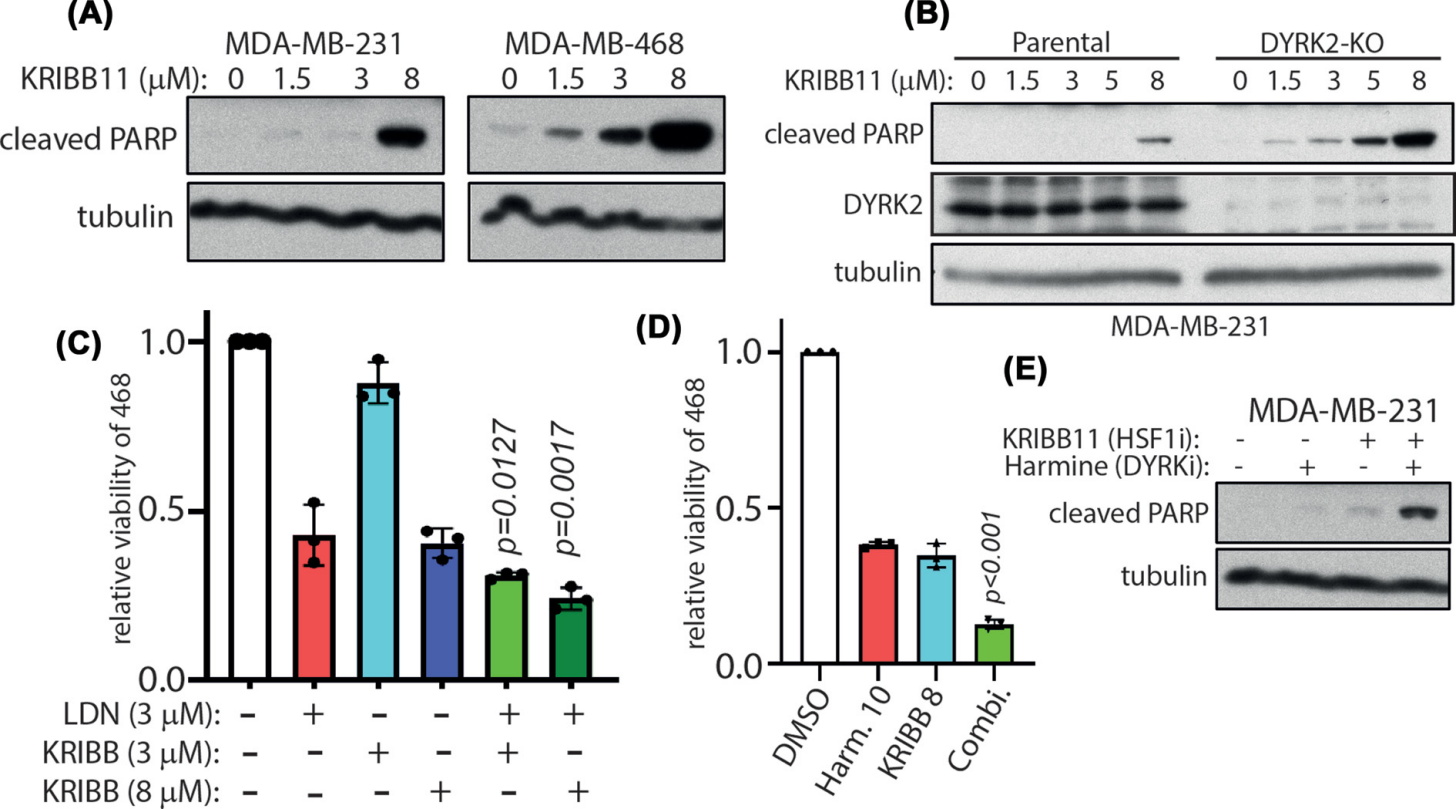
Dual loss of HSF1 and DYRK2 induces enhanced apoptosis in TNBC cells (**A**) MDA-MB-231 and MDA-MB-468 cells were treated with or without the indicated concentrations of KRIBB11 for 16 h. Cells were lysed and immunoblotting was carried out with the indicated antibodies. (**B**) MDA-MB-231 parental or DYRK2 knock-out cells were treated with or without the indicated concentration of KRIBB11 for 24 h. Cells were lysed and immunoblotting was carried out with the indicated antibodies. (**C**) MDA-MB-468 cells were treated with either LDN192960 alone or KRIBB11 alone or the combination of both at the indicated concentrations for 72 h and cell viability was analysed by CellTiter 96® AQueous Non-Radioactive Cell Proliferation Assay kit. Data are represented as relative viability of DMSO-treated control. (The *P*-value provided is the least significant value comparing the combination vs. single-drug treatments. Two-way ANOVA with multiple comparison: Fisher’s LSD test). (**D**) MDA-MB-468 cells were treated with either Harmine alone or KRIBB11 alone or the combination of both at the indicated concentrations for 72 h and cell viability was analysed as in (C). (**E**) MDA-MB-231 parental cells were treated with or without 3 μM KRIBB11 and/or 10 μM harmine for 24 h. Cells were lysed and immunoblotting was carried out with the indicated antibodies.

It is well established that HSF1 and DYRK2 are individually viable targets in TNBC. Hence, our next aim was to determine if dual inhibition could induce cytotoxicity synergistically/additively *in vitro*. TNBC MDA-MB-468 and MDA-MB-231 cells were treated with a HSF1 inhibitor, KRIBB11, at the indicated concentrations and a western blot was performed to measure apoptotic cell death by the accumulation of cleaved parp (Figure 2A). Indeed at 8 μM, significant accumulation of cleaved PARP was observed suggesting that KRIBB11 induces cell death via apoptosis in TNBC cells (Figure 2A). In a previous study, we had generated MDA-MB-231 cells with a Crispr/Cas9 mediated deletion of DYRK2 (DYRK2-KO) [14]. We treated parental or DYRK2-KO MDA-MB-231 cells with varying concentrations of KRIBB11 and observed that DYRK2-KO cells exhibited significant increase in cleaved PARP at a lower KRIBB11 concentration than parental cells (Figure 2B). Next, to explore if a pharmacological combination of KRIBB11 with DYRK2 inhibitors could additively induce cell death in TNBC, MDA-MB-468 cells treated with a combination of DYRK2 inhibitor LDN192960 and KRIBB11 exhibit a moderate but statistically significant improvement in sensitivity compared with individual LDN192960 or KRIBB11 treatments. This sensitivity was consistent between two concentrations of KRIBB11 3 and 8 µM in combination with 3 µM LDN192960 (Figure 2C). Furthermore, MDA-MB-468 and MDA-MB-231 cells were treated with DMSO vehicle, DYRK2 inhibitor harmine or KRIBB11 individually or in combination (Figure 2D,E). The combination of harmine and KRIBB11 has a much stronger cell death induction (Figure 2D) accumulation of cleaved PARP (Figure 2E), suggesting an increased induction of apoptosis compared with individual drug treatments.

### Dual inhibition of DYRK2 and HSF1 sensitises proteasome-inhibitor-resistant cells

**Figure 3:**
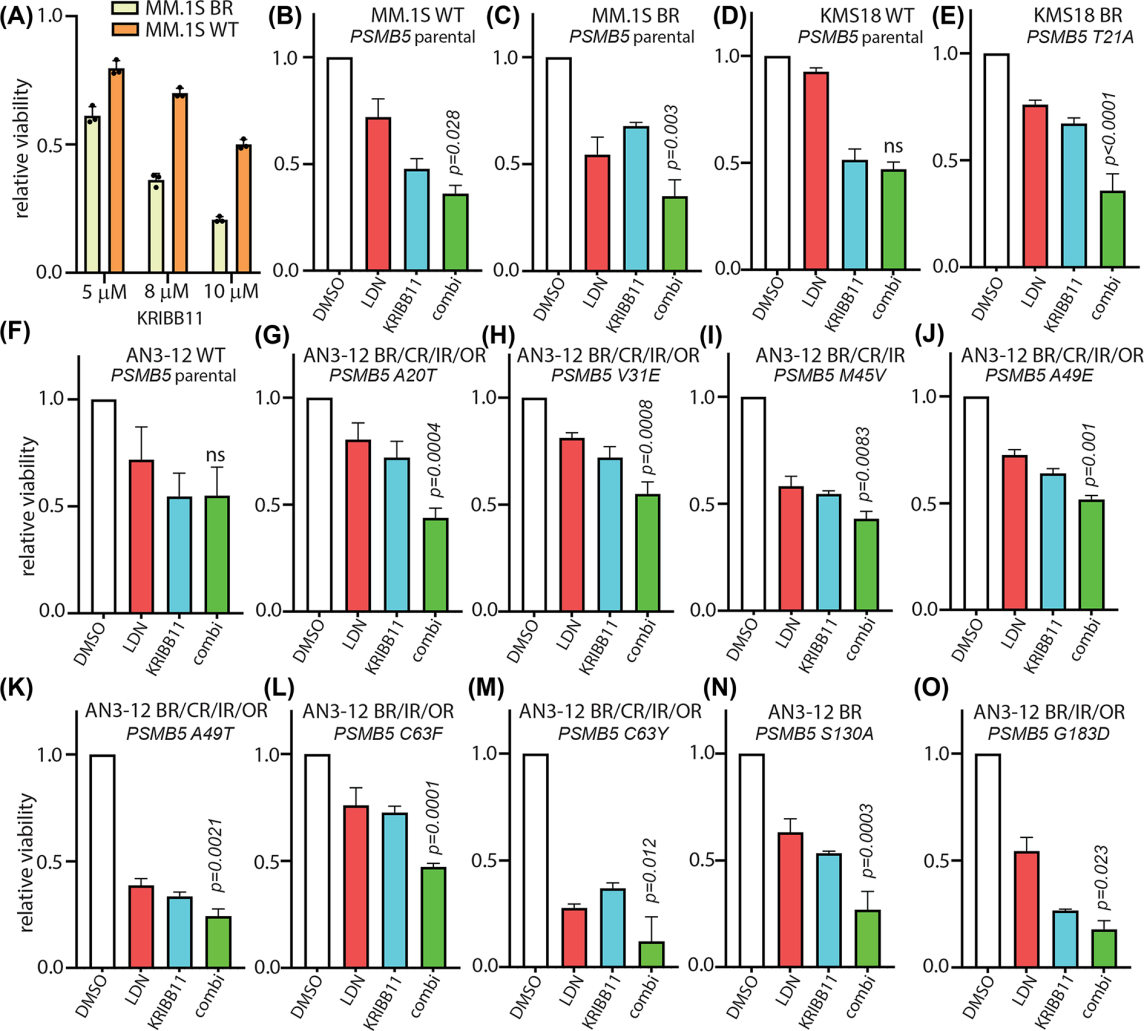
Dual inhibition of HSF1 and DYRK2 bypasses proteasome-inhibitor resistance (**A**) MM.1S parental and MM.1S BR cells were treated with or without the indicated concentrations of KRIBB11 for 72 h and cell viability was analysed by CellTiter 96® AQueous Non-Radioactive Cell Proliferation Assay kit. Data are represented as relative viability of DMSO-treated control. (**B**) MM.1S parental cells were treated with either 5 μM LDN192960 alone or 8 μM KRIBB11 alone or the combination of both for 72 h and cell viability was analysed by CellTiter 96® AQueous Non-Radioactive Cell Proliferation Assay kit. Data are represented as relative viability of DMSO-treated control. (**C**) MM.1S BR cells were treated with either 5 μM LDN192960 alone or 5 μM KRIBB11 alone or the combination and analysed as in (B). (**D**) KMS18 parental cells were treated with either 3 μM LDN192960 alone or 8 μM KRIBB11 alone or the combination and analysed as in (B). (**E**) KMS18 T21A cells were treated with either 8 μM LDN192960 alone or 5 μM KRIBB11 alone or the combination and analysed as in (B). (**F**) AN3-12 parental cells were treated with either 5 μM LDN192960 alone or 5 μM KRIBB11 alone or the combination and analysed as in (B). (**G**) AN3-12 A20T cells were treated with either 5 μM LDN192960 alone or 3 μM KRIBB11 alone or the combination and analysed as in (B). (**H**) AN3-12 V31E cells were treated with either 5 μM LDN192960 alone or 3 μM KRIBB11 alone or the combination and analysed as in (B). (**I**) AN3-12 M45V cells were treated with either 3 μM LDN192960 alone or 3 μM KRIBB11 alone or the combination and analysed as in (B). (**J**) AN3-12 A49E cells were treated with either 3 μM LDN192960 alone or 3 μM KRIBB11 alone or the combination and analysed as in (B). (**K**) AN3-12 A49T cells were treated with either 3 μM LDN192960 alone or 5 μM KRIBB11 alone or the combination and analysed as in (B). (**L**) AN3-12 C63F cells were treated with either 3 μM LDN192960 alone or 3 μM KRIBB11 alone or the combination and analysed as in (B). (**M**) AN3-12 C63Y cells were treated with either 10 μM LDN192960 alone or 3 μM KRIBB11 alone or the combination and analysed as in (B). (**N**) AN3-12 S130A cells were treated with either 10 μM LDN192960 alone or 10 μM KRIBB11 alone or the combination and analysed as in (B). (**O**) AN3-12 G183D cells were treated with either 8 μM LDN192960 alone or 8 μM KRIBB11 alone or the combination and analysed as in (B). See also Supplementary Figure S2 for further establishment of drug sensitivities of AN3-12 PSMB5 mutant cells. The *P*-value provided is the least significant value comparing the combination vs. single-drug treatments; ns: not significant (two-way ANOVA with multiple comparison: Fisher’s LSD test). Abbreviations: BR, bortezomib resistant; CR, carfilzomib resistant; IR, ixazomib resistant; OR, oprozomib resistant.

Since TNBC cells are sensitive to dual inhibition of DYRK2 and HSF1, we next explored if proteasome-inhibitor-resistant cell lines could also be targeted by the dual pharmacological inhibition. We have previously shown that bortezomib-resistant cell MM.1S BR is sensitive to DYRK2 inhibition both *in vitro* and *in vivo* [15,16]. MM.1S BR cells do not harbour any PSMB5 mutations and hence is expected to be dependent on HSF1 transcriptional activity for survival. Indeed, MM1S.BR cells were more sensitive to KRIBB11 as compared with parental MM.1S cells (Figure 3A) and were significantly more sensitive to LDN192960 and KRIBB11 combined compared with the parental MM.1S (Figure 3B,C). To further explore the effect of LDN192960 and KRIBB11 combination treatment, we observed that bortezomib-resistant KMS18 cells harbouring PSMB5 T21A mutation were similarly more sensitive to the combination than parental KMS18 cells (Figure 3D,E). As expected, murine haploid stem cell line AN3-12 parental did not exhibit significant sensitivity to LDN192960 and KRIBB11 combination (Figure 3F); however, proteasome-inhibitor-resistant PSMB5 mutant knock-in AN3-12 cells exhibited varying degrees of sensitivity to the combination. AN3-12 cells with PSMB5 knock-in mutations exhibit a varying degree of resistance to different proteasome inhibitors yet the mutants A20T (Figure 3G), V31E (Figure 3H), M45V (Figure 3I), A49E (Figure 3J), A49T (Figure 3K), C63F (Figure 3L), C63Y (Figure 3M), S130A (Figure 3N), and G183D (Figure 3O) exhibited moderate to modest but statistically significant enhanced sensitivity to DYRK2 and HSF1 dual inhibition. PSMB5 G183D-mutated AN3-12 cells is a novel mutation not reported previously and it exhibits resistance to 10 nM bortezomib (BR), 50 nM ixazomib (IR), and 80 nM oprozomib (OR) but is sensitive to 15 nM carfilzomib (Supplementary Figure S2A). Interestingly, V31G-mutated cells did not exhibit any enhanced sensitivity to the dual inhibition (Supplementary Figure S2B). In fact, the different mutants exhibited varied sensitivities to individual 3 μM treatments of KRIBB11 or LDN192960 (Supplementary Figure S2C,D). Together, the data suggest that dual pharmacological targeting of HSF1 and DYRK2 could induce death in proteasome-inhibitor-resistant cells.

### DYRK2 nuclear levels predict cancer recurrence

**Figure 4:**
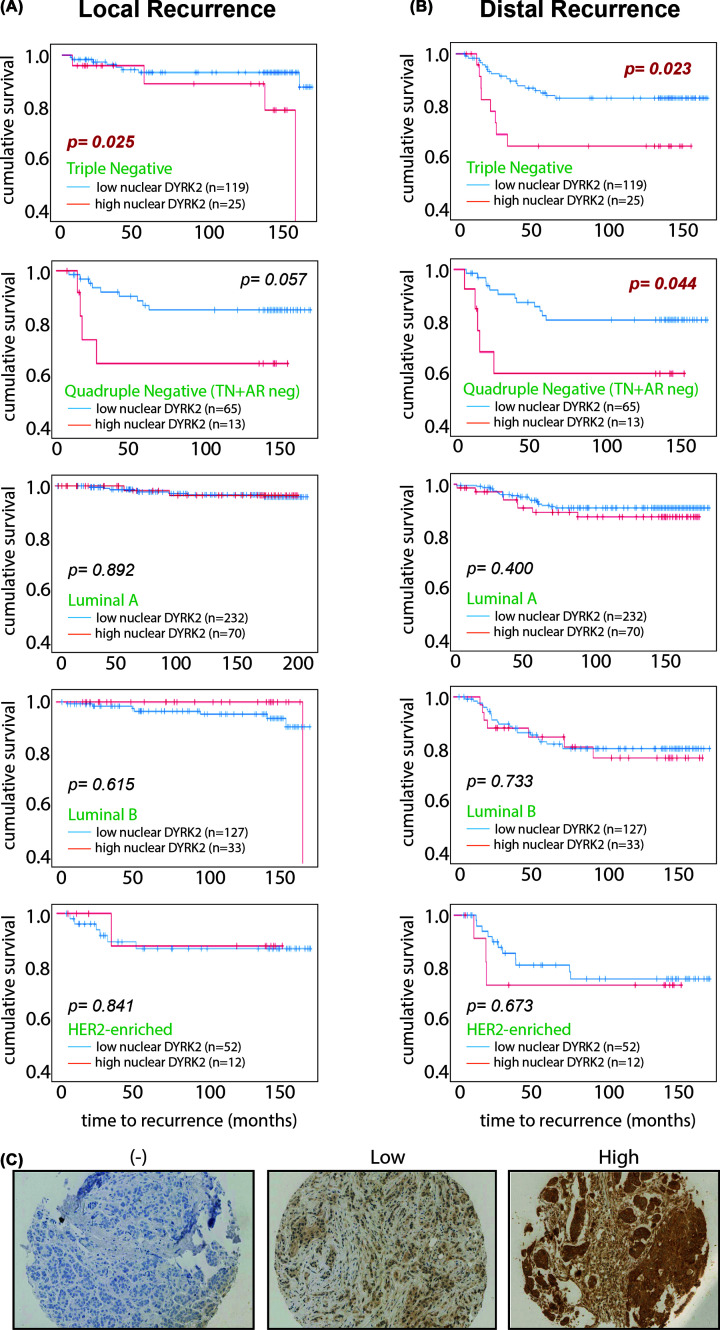
High nuclear DYRK2 expression predicts faster TNBC recurrence Relationship between nuclear DYRK2 levels in tumour cells and time to (**A**) local recurrence or (**B**) distal recurrence in patients with indicated subtypes of breast invasive ductal carcinoma. Data represented as Kaplan–Meier curves and *P*-value were derived from survival curve comparison using Mantel–Cox Log-rank test. (**C**) Representative photomicrographs of tumours from the tissue microarray that were stained by DYRK2 IHC and scored as having either no (−), low, or high nuclear DYRK2 expression.

We have previously shown that nuclear expressions of DYRK2 and HSF1 correlate in TNBC and quadruple-negative breast cancer (QNBC: ER, PR, HER2, and androgen receptor negative) subtypes of invasive ductal carcinoma patient tumours. Interestingly, nuclear DYRK2 protein expression correlates with shorter TNBC local (Figure 4A) and TNBC and QNBC distal recurrence times (Figure 4B). Out of the 850 breast cancer samples on the tissue microarray, this was only observed in the TNBC and QNBC cohorts. These results establish DYRK2 as a potential prognostic factor and promising novel therapeutic target in TNBC, especially in the QNBC subgroup of patients, for whom there is no targeted therapy available. It is also important to note that MDA-MB-231 cells were identified previously as quadruple negative with no androgen receptor expression [28]. Thus, there is full agreement between our cell culture and tissue analysis data.

### The DYRK2-HSF1 axis promotes tumour growth

**Figure 5:**
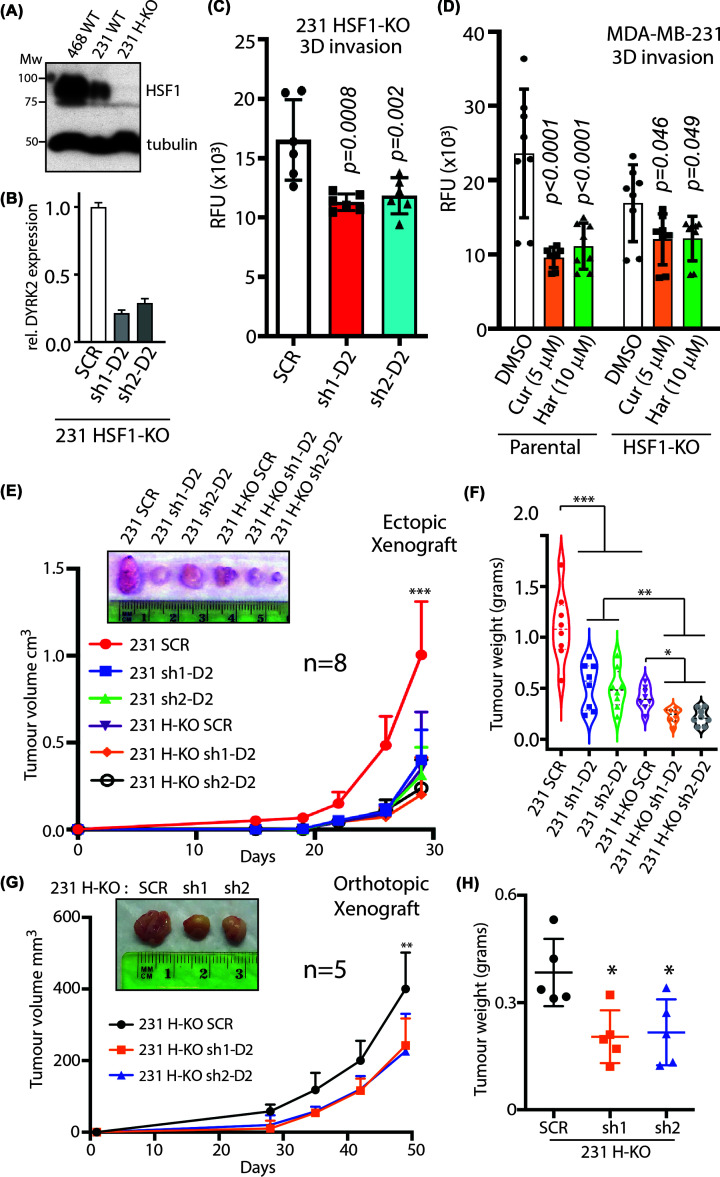
Dual depletion of DYRK2 and HSF1 impedes tumour growth *in vivo* (**A**) Immunoblot confirming Crispr/Cas9-mediated H-KO MDA-MB-231 cells. (**B**) Quantitative PCR analysis to confirm shRNA-mediated DYRK2 knock-down in H-KO MDA-MB-231 cells. (**C**) Bar graph depicting cell invasion in a Matrigel transwell migration assay using MDA-MB-231 H-KO cells with the indicated shRNA. Data were acquired 18 h after seeding in upper chamber of 8 μm pore size transwells. Cells that invaded the Matrigel were quantified based on DNA content using CyQuant dye and data represented as RFU (relative fluorescence units). Reported *P*-value is derived by comparing to H-KO SCR cells, two-way ANOVA, mean ± SD from *n*=2 independent experiments with triplicates in each. (**D**) Bar graph depicting cell invasion in a Matrigel transwell migration assay using DMSO treated or 5 μM curcumin or 10 μM harmine-treated MDA-MB-231 parental or H-KO cells. Data were acquired as in (C). Reported *P*-value is derived by comparing to DMSO-treated control cells, two-way ANOVA, mean ± SD, with Fisher’s LSD multiple comparison from *n*=2 independent experiments with triplicates in each. (**E**) A total of 300000 MDA-MB-231 cells with or without the indicated genome editing or shRNA load were injected subcutaneously in NSG mice. Tumour volume was measured twice a week (*n*=8 mice per condition) and growth curves were plotted. ***P<0.001 (compared with parental group, two-way ANOVA, mean ± SD with Tukey’s multiple comparison). (**F**) Tumours from (A) were resected and tumour weight was measured. ***P<0.001, **P<0.01, *P<0.05 (ordinary one-way ANOVA, mean ± SD with Kruskal–Wallis multiple comparison from *n*=8 mice each). (**G**) A total of 300000 MDA-MB-231 HSF1 KO cells with the indicated shRNA load were injected into the mammary-fat pad of J:NU nude mice. Tumour volume was measured twice a week (*n*=5 mice per condition) and growth curves were plotted. **P<0.01 (compared with parental group, two-way ANOVA, mean ± SD with Tukey’s multiple comparison). (**H**) Tumours from (C) were resected and tumour weight was measured. *P<0.05 (ordinary one-way ANOVA, mean ± SD with Kruskal–Wallis multiple comparison from *n*=5 mice each).

We have previously shown that loss of DYRK2 expression and function significantly reduce 3D TNBC invasion through a matrigel matrix [15,16]. Moreover, the data from breast cancer tissue suggest that nuclear DYRK2 expression could promote breast cancer invasiveness and recurrence. Hence, we utilised our HSF1 knock-out (H-KO) MDA-MB-231 cells (Figure 5A) and further used two independent shRNAs to knock-down DYRK2 (Figure 5B) and carried out an invasion assay. H-KO cells bearing shRNA targetting DYRK2 exhibited significantly lower 3D invasion through a Matrigel (Figure 5C). This was also observed in H-KO cells treated with curcumin and harmine wherein, loss of DYRK2 in H-KO cells induced further reduction in invasive potential in MDA-MB-231 TNBC cell line (Figure 5D).

Based on our cell culture and tissue data, we wondered whether targeting this newly identified DYRK2-HSF1 link could affect tumour growth *in vivo*. To answer this question, we evaluated the tumour formation capacity of MDA-MB-231 parental and HSF1-KO TNBC cells after DYRK2 knock-down by shRNA as stated previously. Tumour volume was measured at the indicated time points, and after MDA-MB-231 parental-derived tumour reached the approximate volume of 1.2 cm^3^, the mice were killed, tumours resected, and the weight of the tumours were measured. Both tumour volume and tumour weight were significantly lower in the H-KO cells compared with parental (Figure 5E,F) which is consistent with the previous literature. Similarly, DYRK2 knock-down cell-derived tumours were smaller in volume and weight than parental as well (Figure 5E,F), which is consistent with our own previous work. Interestingly, tumours derived from H-KO cells bearing shRNAs against DYRK2, exhibited a statistically significant reduction in tumour weight compared with all scrambled control cells (parental and H-KO) (Figure 5F). Since tumours derived from H-KO cells grew much more slowly than those from parental cells, it was difficult to unambiguously conclude the effect of a further DYRK2 depletion on tumour growth in H-KO cells. To explore this further and to delineate the role of dual inhibition, we utilised the scrambled control or DYRK2 knock-down cells in the H-KO MDA-MB-231 background alone. We generated an orthotopic mammary-fat pad-derived breast cancer model in athymic nude mice and observed tumour growth. Indeed, tumour volume and tumour weight were significantly lower in the H-KO cells bearing shRNA-targeting DYRK2 compared with scrambled control (Figure 5G,H). This clearly suggests that dual inhibition of DYRK2 and HSF1 will impede tumour growth *in vivo* at an enhanced rate compared with individual targeting. These experiments illustrate the importance of both DYRK2 and HSF1 for TNBC tumour growth and further show that DYRK2 plays a major role in the growth of HSF1-proficient tumours. Overall, our data support the potential biological importance of the DYRK2-HSF1 axis in regulating cancer cell growth *in vivo*.

## Discussion

Our current work proposes a novel mechanism of targeting malignancies via perturbing upstream regulators of the stress adaptation and chemoresistance induction pathways. We had previously established DYRK2 as a direct HSF1 phosphorylating kinase and showed that DYRK2 inhibition leads to impediment of the cell cycle via accumulation of proapoptotic factors leading to tumour regression of multiple myeloma and TNBC *in vivo*. Initially, we had thought that this pathway was specific primarily for TNBC and myeloma; however, this work shows that HSF1 and DYRK2 expressions correlate across cell states (Figure 1A–D) and therapy responses (Figure 1E,F) in diverse cancer types. In fact, expressions of HSF1 and DYRK2 seem to cluster together during chemotherapy response or at predicting response to cancer monotherapies (Figure 1E,F). We had observed this correlation previously at protein levels wherein nuclear DYRK2 expression positively correlated with nuclear HSF1 and this dual expression predicted poor patient prognosis in TNBC tumour samples. Hence, DYRK2 targeting in various cancers have been gaining traction over the last 5 years since our work asserted the protumourigenic role of the kinase. Various small-molecule inhibitors have been developed targeting the DYRK kinases [12]. LDN192960 is a pan-DYRK inhibitor but it actively inhibits proteasome activity in cells, *in vivo*, and synergises with proteasome inhibitors in inducing cytotoxicity in cancer-specific manner. Furthermore, LDN192960 sensitises bortezomib-resistant myeloma cells as well [16]. Bortezomib-resistance in myeloma occurs either due to up-regulation of HSF1 activity or due to altered redox homeostasis [29] or, in rare cases, due to accumulation of mutations in key bortezomib-docking sites in proteasome subunit of PSMB5 [8]. Although PSMB5 mutations are largely thought to perturb drug docking, recent evidence suggests that some of those mutations exhibit markedly reduced proteasome activities as well [21], suggesting potential up-regulation of HSF1 pathway. This further adds traction to our hypothesis that pharmacological inhibition of HSF1 in combination with DYRK2 inhibitors could induce enhanced cytotoxicity in cancer cells while also sensitise bortezomib-resistant cells. The HSF1 pathway represents an attractive therapeutic target as it plays an important role in cancer initiation and in cancer progression and chemoresistance. Furthermore, higher expression of HSF1 predicts poor progression-free survival in diverse cancers [30,31]. Indeed, the HSF1 inhibitor KRIBB11 induces apoptosis in TNBC cells (Figure 2A). Consistent with our hypothesis, KRIBB11 induces apoptosis at a much lower dose in DYRK2 null TNBC cells (Figure 2B), while a combination of DYRK2 inhibitor and KRIBB11 induces enhanced apoptotic death in TNBC cells (Figure 2C–E). Interestingly, the combination of LDN192960 and KRIBB11 induces significantly more cytotoxicity in proteasome-inhibitor-resistant cells (Figure 3). Most PSMB5-mutated cells exhibit marked resistance to proteasome inhibitors coupled to loss of chymotryptic-like activities to varied degrees [21], leading to diverse sensitivities to KRIBB11 or LDN192960 monotherapies (Supplementary Figure S2C,D). Intriguingly, PSMB5 mutation at V31G does not exhibit sensitivity to combined inhibition of HSF1 and DYRK2 (Supplementary Figure S2B). This is likely since V31G preserves complete chymotryptic-like proteasome activity, exhibits modest resistance to bortezomib, is parental-like sensitive to carfilzomib, oprozomib, KRIBB11, and LDN192960, and is the most neutral PSMB5 mutation observed in our hands [21]. Surprisingly, G183D mutant exhibited more resistance to both HSF1 and DYRK2 inhibitor monotherapies than the other mutants (Supplementary Figure S2C,D) but was sensitive to a combined treatment (Figure 3O). This suggests that the drug combination could promote yet unknown pleiotropic effects in specific resistant cells beyond proteotoxicity, which deserves more analysis in the future. Proteasome-inhibitor resistance is extensively observed in multiple myeloma patients and hence dual targeting of HSF1 and DYRK2 could be an alternative strategy to combat the refractory disease.

Previously, we had shown that DYRK2 regulates the nuclear localisation of HSF1 and indeed nuclear expressions of DYRK2 and HSF1 correlates in TNBC and QNBCs poor outcome and time to recurrence. Here, we show that higher nuclear DYRK2 levels directly correlates with shorter local and distal time to recurrence in TNBC and QNBC (Figure 4). Indeed, loss of both HSF1 and DYRK2 led to reduced 3D matrigel invasion (Figure 5A–D) and tumour burden in both ectopic and orthotopic QNBC tumour xenograft (Figure 5E–H). Hence, the DYRK2-HSF1 pathway represents an attractive therapeutic target since it plays an important role in cancer progression and chemoresistance. Although much work is needed to develop *in vivo* potent and clinically relevant DYRK2 inhibitors, the work does endorse cotargetting of a kinase and transcription factor as a viable therapeutic option, especially in hard-to-treat breast cancer subtypes and drug-resistant refractory myeloma with a good potential of expanding to other cancers with unmet need.

## Supplementary Material

Supplementary Figures S1-S2

## Data Availability

All data that support the findings of the present study are included in this manuscript. Further information and reagents are available upon request to the corresponding author, S.B.

## Open Access

Open access for this article was enabled by the participation of University of Dundee in an all-inclusive *Read & Publish* agreement with Portland Press and the Biochemical Society under a transformative agreement with JISC.

## Abbreviations

BR: bortezomib resistant
BSA: bovine serum albumin
DYRK2: Dual specificity tyrosine phosphorylation-regulated kinase 2
FBS: fetal bovine serum
H-KO: HSF1 knock-out
HSF1: heat-shock factor 1
IR: ixazomib resistant
OR: oprozomib resistant
QNBC: quadruple-negative breast cancer
sc-RNAseq: single-cell RNA sequencing
TMA: tissue microarray
TNBC: triple-negative breast cancer
t-SNE: t-distributed stochastic neighbour embedding
uMAP: uniform manifold approximation and projection
