# Supplementary material for: Dual inhibition of HSF1 and DYRK2 impedes cancer progression
Source: Biosci Rep. 2023 Jan 30;43(1):BSR20222102. doi: 10.1042/BSR20222102 (PMC9894012; doi:10.1042/BSR20222102)
Supplement: Supplementary Figures S1-S2 [file BSR-2022-2102_supp.pdf]

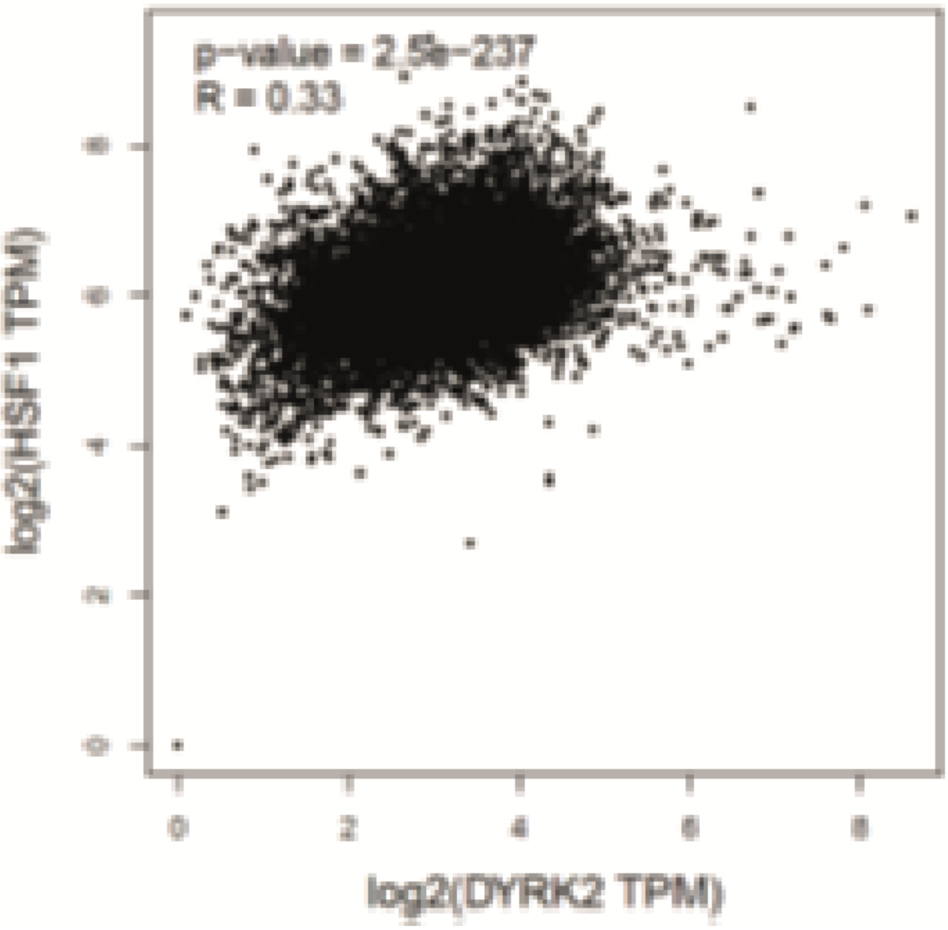

Supplementary Figure S1: DYRK2 and HSF1 mRNA expressions positively correlate in the cancer genome atlas database. The overall expression of HSF1 and DYRK2 were correlated across all cancers in the TCGA database using GEPIA webtool. P-value and Spearman’s R were calculated trough GEPIA and provided in the figure.

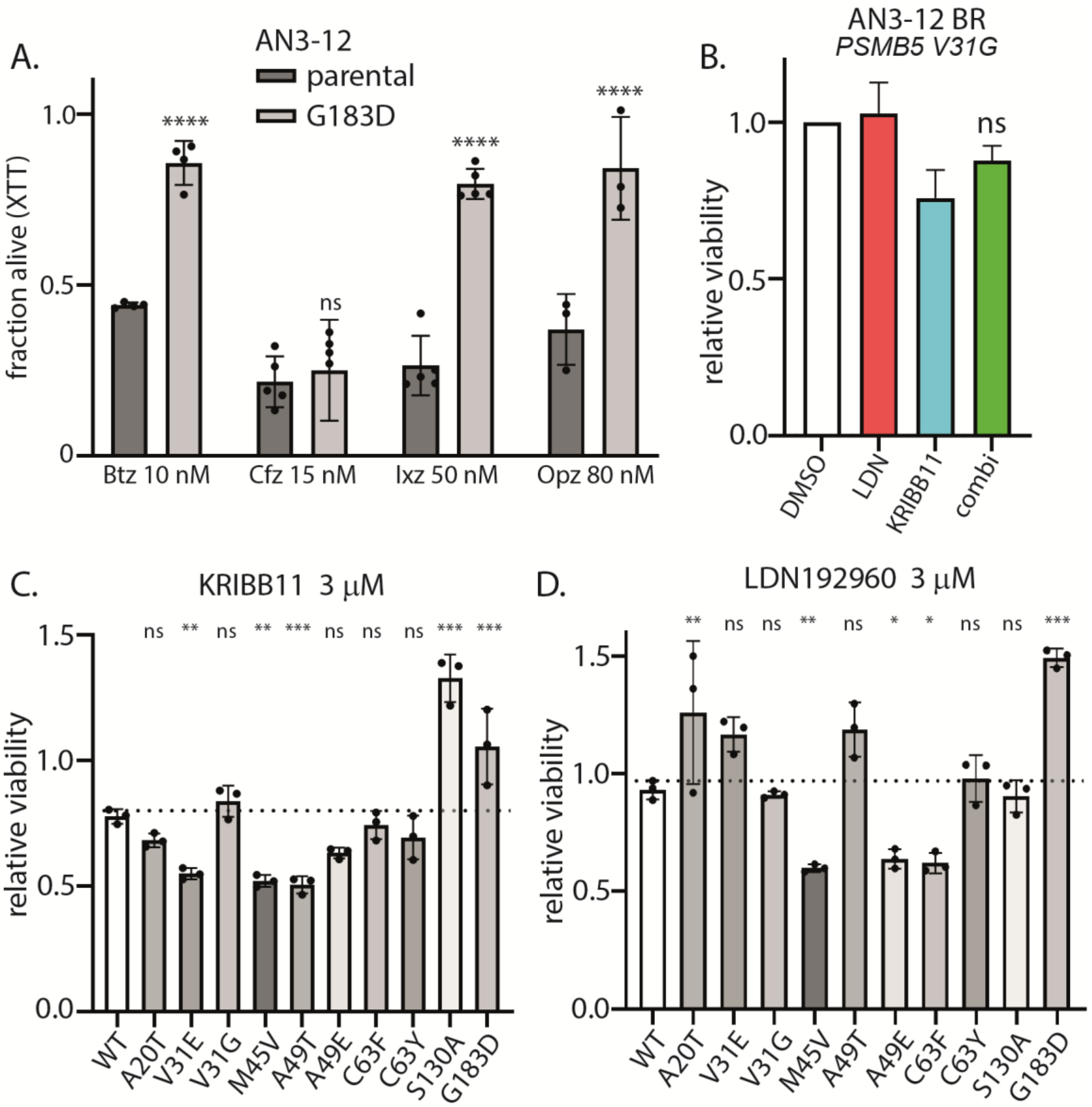
A.
AN3-12
parental
G183D
fraction alive (XTT)
1.0
0.5
0
****
ns
****
****
Btz 10 nM
Cfz 15 nM
Ixz 50 nM
Opz 80 nM
B.
AN3-12 BR
PSMB5 V31G
relative viability
1.0
0.5
0.0
ns
DMSO
LDN
KRIBB11
combi
C.
KRIBB11 3 μM
relative viability
1.5
1.0
0.5
0.0
ns ** ns ** *** ns ns ns *** ***
WT
A20T
V31E
V31G
M45V
A49T
A49E
C63F
C63Y
S130A
G183D
D.
LDN192960 3 μM
relative viability
1.5
1.0
0.5
0.0
** ns ns ** ns * * ns ns ***
WT
A20T
V31E
V31G
M45V
A49T
A49E
C63F
C63Y
S130A
G183D

Supplementary Figure S2: Establishing drug sensitivity of AN3-12 PSMB5 mutant cells.

(A) Cell viability assay (XTT) of AN3-12 parental or isolated PSMB5 G183D bearing clones treated with indicated concentrations of proteasome inhibitors for 48 hrs. ****$p<0.0001$; ns: not significant (one-way ANOVA mean ± SD with Tukey's multiple comparison). Btz: bortezomib; Cfz: carfilzomib; Ixz: ixazomib; Opz: oprozomib.

(B) AN3-12 V31G cells were treated with either 5 μM LDN192960 alone or 3 μM KRIBB11 alone or the combination of both for 72 hr and cell viability was analysed by CellTiter 96® AQueous Non-Radioactive Cell Proliferation Assay kit. Data is represented as relative viability of DMSO-treated control.

(C) Cell viability assay (MTS) of wild-type (WT) control cells and PSMB5 mutated clones treated with 3 μM KRIBB11 for 72 hr. Statistical significance was calculated by one-way ANOVA Dunnett's post-hoc test. ***$p < 0.001$, **$p < 0.01$, *$p < 0.05$, ns not significant. Mean + SD ($n = 3$).

(D) Wild-type control cells and PSMB5 mutated clones treated with 3 μM LDN192960 for 72 hr and data analysed as in (C).
